# Cone-beam computed tomography with automated bone subtraction in preoperative embolization for pelvic bone tumors

**DOI:** 10.1371/journal.pone.0175907

**Published:** 2017-04-18

**Authors:** Dae Yong Park, Hyo-Cheol Kim, Jin Wook Chung, Saebeom Hur, Minuk Kim, Myungsu Lee, Hwan Jun Jae

**Affiliations:** Department of Radiology, Seoul National University Hospital, Seoul, Korea; North Shore Long Island Jewish Health System, UNITED STATES

## Abstract

**Purpose:**

To evaluate the usefulness of cone-beam computed tomography with automated bone subtraction (CBCT-ABS) in the preoperative embolization of hypervascular tumors located in the pelvic bone.

**Materials and methods:**

This retrospective study included 26 patients with pelvic bone tumors who underwent preoperative embolization between January 2014 and October 2016. A CBCT-ABS scan was taken in a total of 17 patients (CBCT-ABS group), and only a series of digital subtraction angiographies (DSAs) was taken in the remaining 9 patients (DSA group). The percent devascularization, number of angiographic runs, total dose-area product (DAP), fluoroscopy time, interventional procedure time, operative time, and estimated blood loss were compared between the two groups using Mann-Whitney test.

**Results:**

The percent devascularization, interventional procedure time, fluoroscopy time, operative time, and estimated blood loss were not statistically different between the two groups (p > 0.05). On the other hand, the number of angiographic runs in the CBCT-ABS group was significantly lower than that in the DSA group (p = 0.029). The total DAP of the CBCT-ABS group (mean, 17700.7 μGym^2^) was higher than that of the DSA group (mean, 8939.4 μGym^2^) (p = 0.002).

**Conclusions:**

The use of CBCT-ABS during the preoperative embolization of pelvic bone tumors significantly reduces the number of angiographic runs at the cost of an increased radiation dose.

## Introduction

As the incidence of hypervascular tumors that often metastasize to bones is increasing around the world, the importance of preoperative embolization is continuing to gather recognition among orthopedic surgeons and interventional radiologists [1–3]. Preoperative intra-arterial embolization has been credited with reducing intraoperative blood loss and is performed in patients with hypervascular tumors prior to surgery in many hospitals [4–10].

With the advent of C-arm angiography merged with flat-panel detectors, more widely known as cone-beam computed tomography (CBCT), it has become possible to obtain rotational images during intervention that can then be reconstructed to create cross-sectional images like those seen in conventional computed tomography. Moreover, the volumetric data can also be used to produce a three-dimensional reconstruction image of the arteries and tumor, allowing the interventional radiologist to visualize important structures more vividly and grasp the spatial relationship of the arterial branches more fully. These advantages of CBCT over the digital subtraction angiography (DSA) triggered its application in diverse interventional procedures, including chemoembolization, radioembolization, percutaneous biopsy, abscess drainage, and adrenal vein sampling [11–15].

However, even with CBCT, the preoperative embolization of tumors located especially in the pelvic bone remains a troublesome task because large bony structures interfere with the visibility of the tumor feeders that are already plentiful, intricate, and tortuous. If all the bone and neighboring structures can be digitally subtracted in the three-dimensional reconstruction image, leaving just the arteries and hypervascular tumor, preoperative embolization might be facilitated. Hence, this study aimed to evaluate the usefulness of CBCT with automated bone subtraction (CBCT-ABS) in the preoperative embolization of tumors located in the pelvic bone.

## Materials and methods

### Patients

The Institutional Review Board approved this retrospective study, and informed consent from the patient was waived because of the retrospective nature of this study (IRB of Seoul National University Hospital, Approval number, 1602-065-740). From January 2014 to October 2016, a total of 69 patients with skeletal tumors underwent preoperative embolization in our institution. Inclusion criteria were 1) tumors in the pelvic bone, 2) preoperative embolization of a pelvic bone tumor, 3) subsequent surgery within two days after embolization, and 4) using angiographic equipment of Siemens. Among 69 patients, 26 patients met the inclusion criteria. The study population consisted of 19 men and 7 women (age range, 12–84 years; mean age, 50.5 years). A CBCT-ABS scan along with DSA was taken in a total of 17 patients (CBCT-ABS group), whereas only a series of DSAs was taken in the remaining 9 patients (DSA group) (Table 1).

**Table 1:** Clinical characteristics of 26 patients.

Variables		Total (n = 26)	DSA (n = 9)	CBCT-ABS (n = 17)	P-value
Age (yr±SD)		50.5±19.0	50.2±18.2	50.7±19.1	0.94
Sex	Male	19 (73%)	6 (67%)	13 (76%)	0.66
	Female	7 (27%)	3 (33%)	4 (24%)	
Tumor location	Right	12 (46%)	5 (56%)	7 (41%)	0.68
	Left	14 (54%)	4 (44%)	10 (59%)	
Tumor size (cm±SD)		9.8±4.2	9.3±4.1	10.1±3.9	0.67
Type of tumor	Hepatocellular Carcinoma	8 (31%)	3 (33%)	5 (29%)	
	Renal Cell Carcinoma	4 (15%)	2 (22%)	2 (12%)	
	Osteosarcoma	4 (15%)	1 (11%)	3 (18%)	
	Chondrosarcoma	3 (12%)	1 (11%)	2 (12%)	
	Other Malignant Tumors^#^	7 (27%)	2(22%)	5 (29%)	

Values are presented as mean± standard deviation or number (%).

^#^Colon cancer, myofibroblastic sarcoma, malignant fibrous histiocytoma, hepatoblastoma, leiomyosarcoma, ovarian cancer, lung cancer

### Imaging system and preoperative embolization

Preoperative embolization was conducted using two fluoroscopic equipments designated for interventional procedures. The patients of the DSA group were treated using a fluoroscopic equipment (AXIOM Artis, Siemens, Erlangen, Germany), and the patients of the CBCT-ABS group were managed using another fluoroscopic equipment (Artis Zee, Siemens). Because the CBCT-ABS has been available since February 2015, all 7 patients who were treated between January 2014 and January 2015 belonged to DSA group. From February 2015, 17 patients were managed by using CBCT-ABS and two by DSA depending on the availability of fluoroscopic equipment.

**Fig 1:**
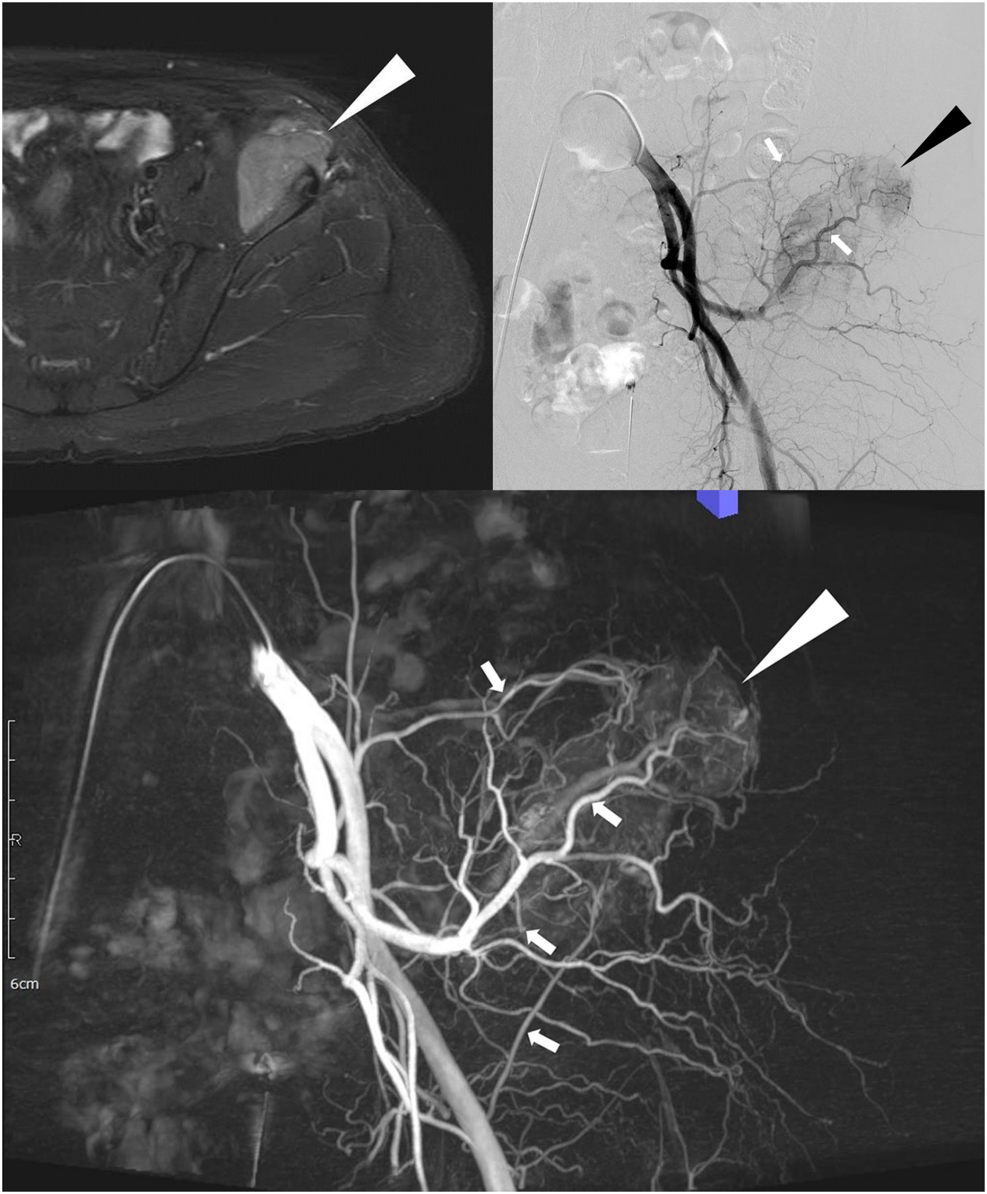
60-year-old man with hepatocellular carcinoma. A. Fat-suppressed contrast-enhanced T1-weighted MR image shows hypervascular tumor (arrowhead) in the left iliac bone. B. The left common iliac angiography shows hypervascular tumor staining (arrowhead) with possible tumor-feeding arteries (arrows): the superior gluteal artery and iliolumbar artery. C. The maximum intensity projection image of the digital subtraction cone-beam CT shows a hypervascular tumor (arrowhead) supplied by four tumor-feeding arteries (arrows): two branches from the superior gluteal artery, one branch of iliolumbar artery, and deep iliac circumflex artery.

Aortogram was first obtained using a 5-Fr pigtail catheter (Cook, Bloomington, IN). In the DSA group, selective angiograms of the internal iliac artery and its branches were obtained to detect tumor-feeding arteries as needed. In the CBCT-ABS group, the CBCT-ABS image was obtained at the internal iliac artery or common iliac artery (Fig 1, S1 & S2 Videos). The parameters for the CBCT-ABS scan are as follows: 0.5° increment, 512 x 512 matrix in projections, total angle of 211°, a total of 419 projections, and a scan time of approximately 7 seconds. Unenhanced CBCT was obtained first, and then an enhanced CBCT image was acquired by injection of contrast media (Pamiray 300, Dongkook Pharmaceutical, Seoul, Korea) 4 seconds before x-ray emission at a flow rate of 3~5 mL/sec for 11 seconds. CBCT images were transmitted to a dedicated workstation (Leonardo with DynaCT, Siemens), where three-dimensional images with automated bone subtraction were reconstructed within 3 minutes.

Based on the findings of the DSA or CBCT-ABS images, tumor-feeding branches were catheterized using microcatheters with a 2.4-Fr tip (Direxion, Boston Scientific, Marlborough, MA) or 2.0-Fr tip (Progreat, Terumo, Tokyo, Japan) and were embolized by polyvinyl alcohol particles (Contour, Boston Scientific) and/or gelatin sponge particles (Cutanplast, MasciaBrunelli, Milano, Italy).

### Data analysis

Radiological and clinical data were retrospectively reviewed by two radiologists (H.C.K. and D.Y.P.) in consensus. Tumor size was recorded as the longest overall diameter with using MR scans. Medical records were reviewed to check the complications related with the procedure such as rectal ischemia or cystitis.

The percent devascularization was estimated by comparing the first DSA and the last DSA taken during the interventional procedure. The number of angiographic runs was determined by counting the number of DSAs taken and adding one if a CBCT-ABS scan was also taken. As for the length of the intervention time, the time elapsed from the puncture of the femoral artery to the removal of the sheath was recorded. The operative time and estimate blood loss (EBL) were calculated from the intraoperative measurements registered by the anesthesiologist. Descriptors of radiation doses, including total dose-area product (DAP) and fluoroscopy time, were automatically measured by all the fluoroscopic equipments.

A total of seven variables, composed of the percent devascularization, number of angiographic runs, total dose-area product, fluoroscopy time, interventional procedure time, operative time, and estimated blood loss, were compared between the two groups using the Mann-Whitney test. The analysis of significance was two-sided, and the statistical significance was set at P value < 0.05. SPSS (SPSS version 21.0, SPSS, Chicago, IL) was used to process and analyze the data.

## Results

The tumor size ranged from 4.0 to 20.4 cm (mean, 9.8 cm). No procedure-related complication was noted in all patients. The percent devascularization was not different between the two groups (p = 0.84) (Table 2). Intervention time, fluoroscopic time, operative time, and estimated blood loss were also not statistically different between the two groups. On the other hand, the number of angiographic runs in the CBCT-ABS group was significantly lower than that in the DSA group (p = 0.029).

**Table 2:** Comparison of preoperative embolization between digital subtraction angiography and cone-beam computed tomography with automated bone subtraction.

Variables	Total (n = 26)	DSA (n = 9)	CBCT-DSA (n = 17)	P-value
	Mean ± SD	Mean ± SD	Mean ± SD	
Percent devascularization (%)	92.5 ± 6.5	91.8 ± 5.7	92.9 ± 7.2	0.84
Intervention time (min)	57.8 ± 20.7	61.7 ± 18.8	55.7 ± 23.6	0.73
Operative time (min)	318.3 ± 138.2	312.2 ± 152.3	321.6 ± 98.6	0.79
Estimated blood loss (mL)	3304 ± 2528	2483 ± 1913	3738 ± 2752	0.24
Number of angiographic runs	5.4 ± 3.0	7.0 ± 4.0	4.5 ± 1.9	0.029
Fluoroscopy time (sec)	940.3 ± 460.4	919.5 ± 243.8	951.2 ± 548.8	0.67
Total dose-area product (μGym^2)	14667.9 ± 8825.8	8939.4 ± 3406.5	17700.7 ± 9363.6	0.002

The total DAP of the CBCT-ABS group (mean, 17700.7 μGym^2^) was significantly higher than that of the DSA group (mean, 8939.4 μGym^2^) (p = 0.002). The mean DAP of one DSA run was 812.7 μGym^2^ and the mean DAP of one CBCT-ABS scan was 10197.2 μGym^2^.

## Discussion

Since its advent in clinical practice, CBCT has become an essential tool in performing both vascular and non-vascular interventional procedures [11–15]. This study investigated the pros and cons of integrating CBCT-ABS in the preoperative embolization of hypervascular tumors located in the pelvic bone. This study focused on tumors located only in the pelvic bone for two main reasons: first, the vascular anatomy in this region is complex, thus multiple DSA runs are frequently obtained to reveal tumor feeders; and, second, the DSA alone was considered sufficient in locating tumor feeders of hypervascular tumors located elsewhere in the body. According to the initial clinical experience presented in this study, the use of CBCT-ABS during preoperative embolization can reduce the number of angiographic runs at the cost of an increased total DAP.

In the conventional preoperative embolization procedure, interventional radiologists use a series of DSA and fluoroscopy images to locate the individual arteries that feed the hypervascular tumor. These two image modalities, however, are two-dimensional and thus display overlapped vasculature that often confuses even the most experienced interventional radiologists in clearly identifying the tumor feeders. To make matters worse, the relatively larger bony structures further complicate the process if the tumor is located in the pelvic bone. CBCT-ABS ameliorated such problems by creating a three-dimensional reconstruction of the vascular system around the tumor and by digitally subtracting nearby obstacles that impaired visibility. As a result, fewer angiographic runs were necessary in order to complete the preoperative embolization. In particular, CBCT-ABS may be more useful for inexperienced operators, and for embolization of large tumors with multiple feeders, and for pelvic bone tumors rather than long bone tumors.

The total DAP of the CBCT-ABS group was significantly greater than that of the DSA group (p = 0.002). When the Siemens fluoroscopic equipment was used, a single CBCT-ABS scan (mean, 10197.2 μGym^2^) emitted a radiation dose equivalent to approximately 12.5 DSAs (mean, 812.7 μGym^2^). Therefore, even though the employment of CBCT-ABS significantly reduced the number of angiographic runs, it did not reduce a radiation dose equivalent by a decrement of 12.5 runs. The number of angiographic runs in the CBCT-ABS group (mean, 4.5) was only about 2.5 fewer than that of the DSA group (mean, 7.0).

The mean total DAP of the CBCT-ABS group was 17700.7 μGym^2^ (177 Gycm^2^), which is within the reference range for patient radiation doses in interventional radiology proposed by Miller et al [16]. Thus, we think that the increased stochastic risk by using CBCT-ABS is negligible in this patient population with bony metastasis. In addition, CBCT can replace a few of angiographic runs, and distribute the skin dose of radiation over a range of 210°, which can reduce the deterministic effects [17]. Unfortunately, cumulative dose is not available in this study, which means that discussion about deterministic effect by CBCT is impossible.

When CBCT-ABS was used, number of DSA runs decreased in this study. However, the procedure time and fluoroscopy time were not reduced with CBCT-ABS. In addition, the use of CBCT-ABS did not influence percent devascularization and the subsequent operation parameters. We think that small study population and more complex cases in CBCT-ABS group may obscure the statistical difference. In addition, complete devascularization of pelvic tumor is commonly impossible due to multiple fine feeding arteries which can not be catheterized, resulting in no difference of operation parameters. Although this study failed to demonstrate the various merits of CBCT-ABS in preoperative embolization of pelvic bone tumor, we think that CBCT-ABC can show us three-dimension configuration of tumor feeders which can facilitate the procedure.

This study contains several drawbacks. First, the study population was relatively small. Thus, generalization of this study results should be proved by further investigation with large study population. Second, this study is retrospective. Thus, the operator’s confidence in defining tumor feeders and impact of CBCT-ABS on decision making during the procedure could not be evaluated. Third, there is a possibility of selection bias. The CBCT-ABS became available on February 2015. After the introduction of CBCT-ABS, complex cases were performed in the equipment with CBCT-ABS, although the usage of CBCT-ABS depended on the availability of the equipment.

In conclusion, the results of this study indicate that the use of CBCT-ABS during the preoperative embolization of hypervascular tumors located in the pelvic bone significantly reduces the number of angiographic runs at the cost of an increased radiation dose.

## Supporting information

S1 Video Digital subtraction rotational angiography of the left common iliac artery. (WMV)

S2 Video A vertical and transverse rotation of the maximum intensity projection image of the digital subtraction cone-beam CT. This three-dimension image shows four tumor-feeding arteries.
(WMV)

## References

[1] Barton PP, Waneck RE, Karnel FJ, Ritschl P, Kramer J, Lechner GL. Embolization of bone metastases. J Vasc Interv Radiol 1996;7: 81–88. 8773979 10.1016/s1051-0443(96)70738-8

[2] Fukutomi M, Yokota M, Chuman H, Harada H, Zaitsu Y, Funakoshi A, et al Increased incidence of bone metastases in hepatocellular carcinoma. Eur J Gastroenterol Hepatol 2001;13: 1083–1088. 11564960 10.1097/00042737-200109000-00015

[3] Owen RJ. Embolization of musculoskeletal bone tumors. Semin Intervent Radiol 2010;27: 111–123. 10.1055/s-0030-1253510 21629401 PMC3036517

[4] Basile A, Rand T, Lomoschitz F, Toma C, Lupattelli T, Kettenbach J, et al Trisacryl gelatin microspheres versus polyvinyl alcohol particles in the preoperative embolization of bone neoplasms. Cardiovasc Intervent Radiol 2004;27: 495–502. 10.1007/s00270-003-0147-1 15383854

[5] Boruban S, Sancak T, Yildiz Y, Saglik Y. Embolization of benign and malignant bone and soft tissue tumors of the extremities. Diagn Interv Radiol 2007;13: 164–171. 17846993

[6] Kickuth R, Waldherr C, Hoppe H, Bonel HM, Ludwig K, Beck M, et al Interventional management of hypervascular osseous metastasis: role of embolotherapy before orthopedic tumor resection and bone stabilization. AJR Am J Roentgenol 2008;191: W240–247. 10.2214/AJR.07.4037 19020210

[7] Kwon JH, Shin JH, Kim JH, Gwon DI, Yoon HK, Ko GY, et al Preoperative transcatheter arterial embolization of hypervascular metastatic tumors of long bones. Acta Radiol 2010;51: 396–401. 10.3109/02841851003660081 20380603

[8] Kobayashi K, Ozkan E, Tam A, Ensor J, Wallace MJ, Gupta S. Preoperative embolization of spinal tumors: variables affecting intraoperative blood loss after embolization. Acta Radiol 2012;53: 935–942. 10.1258/ar.2012.120314 22927661

[9] Kim W, Han I, Jae HJ, Kang S, Lee SA, Kim JS, et al Preoperative embolization for bone metastasis from hepatocellular carcinoma. Orthopedics 2015;38: e99–e105. 10.3928/01477447-20150204-56 25665126

[10] Shimohira M, Nagai K, Hashizume T, Nakagawa M, Ozawa Y, Sakurai K, et al Preoperative transarterial embolization using gelatin sponge for hypervascular bone and soft tissue tumors in the pelvis or extremities. Acta Radiol 2016;57: 457–462. 10.1177/0284185115590435 26082444

[11] Wallace MJ, Kuo MD, Glaiberman C, Binkert CA, Orth RC, Soulez G. Three-dimensional C-arm cone-beam CT: applications in the Interventional Suite. J Vasc Interv Radiol 2009;20: 523–537. 10.1016/j.jvir.2009.04.059 19560037

[12] Park SI, Rhee Y, Lim JS, Park S, Kang SW, Lee MS, et al Right adrenal venography findings correlated with C-arm CT for selection during C-arm CT-assisted adrenal vein sampling in primary aldosteronism. Cardiovasc Intervent Radiol 2014;37: 1469–1475. 10.1007/s00270-013-0820-y 24352864

[13] Kim HC. Role of C-arm cone-beam CT in chemoembolization for hepatocellular carcinoma. Korean J Radiol 2015;16: 114–124. 10.3348/kjr.2015.16.1.114 25598679 PMC4296258

[14] Choi WS, Kim HC, Hur S, Choi JW, Lee JH, Yu SJ, et al Role of C-arm CT in identifying caudate arteries supplying hepatocellular carcinoma. J Vasc Interv Radiol. 2014;25: 1380–1388. 10.1016/j.jvir.2014.02.028 24713418

[15] McKay T, Ingraham CR, Johnson GE, Kogut MJ, Vaidya S, Padia SA. Cone-beam CT with fluoroscopic overlay versus conventional CT guidance for percutaneous abdominopelvic abscess drain placement. J Vasc Interv Radiol 2016;27: 52–57. 10.1016/j.jvir.2015.09.016 26573489

[16] Miller DL, Kwon D, Bonavia GH. Reference levels for patient radiation doses in interventional radiology: proposed initial values for U.S. practice. Radiology 2009;253:753–764. 10.1148/radiol.2533090354 19789226 PMC2786193

[17] Kothary N, Abdelmaksoud MH, Tognolini A, Fahrig R, Rosenberg J, Hovsepian DM, et al Imaging guidance with C-arm CT: prospective evaluation of its impact on patient radiation exposure during transhepatic arterial chemoembolization. J Vasc Interv Radiol 2011;22:1535–1543. 10.1016/j.jvir.2011.07.008 21875814

