# Correction: Cone-beam computed tomography with automated bone subtraction in preoperative embolization for pelvic bone tumors

**DOI:** 10.1371/journal.pone.0181645

**Published:** 2017-07-13

**Authors:** Dae Yong Park, Hyo-Cheol Kim, Jin Wook Chung, Saebeom Hur, Minuk Kim, Myungsu Lee, Hwan Jun Jae

There is an error in the affiliation for all authors. The affiliation should be: Department of Radiology, Seoul National University Hospital, Seoul National University College of Medicine, Seoul, Korea.

## References

[1] Park DY, Kim H-C, Chung JW, Hur S, Kim M, Lee M, et al (2017) Cone-beam computed tomography with automated bone subtraction in preoperative embolization for pelvic bone tumors. PLoS ONE 12(4): e0175907 doi:10.1371/journal.pone.0175907 28419147 10.1371/journal.pone.0175907 PMC5395210

